# Evaluation and integration of disparate classification systems for clefts of the lip

**DOI:** 10.3389/fphys.2014.00163

**Published:** 2014-05-14

**Authors:** Kathie H. Wang, Carrie L. Heike, Melissa D. Clarkson, Jose L. V. Mejino, James F. Brinkley, Raymond W. Tse, Craig B. Birgfeld, David A. Fitzsimons, Timothy C. Cox

**Affiliations:** ^1^Center for Developmental Biology and Regenerative Medicine, Seattle Children's Research InstituteSeattle, WA, USA; ^2^Center for Clinical and Translational Sciences, Seattle Children's Research InstituteSeattle, WA, USA; ^3^Seattle Children's Craniofacial CenterSeattle, WA, USA; ^4^Department of Pediatrics (Division of Craniofacial Medicine), University of WashingtonSeattle, WA, USA; ^5^Department of Biological Structure (Structural Informatics Group), University of WashingtonSeattle, WA, USA; ^6^Department of Biomedical Informatics and Medical Education, University of WashingtonSeattle, WA, USA; ^7^Faculty of Medicine, The Cleft Palate Clinic, The Children's Hospital at Westmead, and Discipline of Paediatrics and Child Health, University of SydneySydney, NSW, Australia; ^8^Department of Anatomy and Developmental Biology, Monash UniversityClayton, VIC, Australia

**Keywords:** cleft lip, classification system, orofacial clefts, forme fruste, incomplete cleft, ontology

## Abstract

Orofacial clefting is a common birth defect with wide phenotypic variability. Many systems have been developed to classify cleft patterns to facilitate diagnosis, management, surgical treatment, and research. In this review, we examine the rationale for different existing classification schemes and determine their inter-relationships, as well as strengths and deficiencies for subclassification of clefts of the lip. The various systems differ in how they describe and define attributes of cleft lip (CL) phenotypes. Application and analysis of the CL classifications reveal discrepancies that may result in errors when comparing studies that use different systems. These inconsistencies in terminology, variable levels of subclassification, and ambiguity in some descriptions may confound analyses and impede further research aimed at understanding the genetics and etiology of clefts, development of effective treatment options for patients, as well as cross-institutional comparisons of outcome measures. Identification and reconciliation of discrepancies among existing systems is the first step toward creating a common standard to allow for a more explicit interpretation that will ultimately lead to a better understanding of the causes and manifestations of phenotypic variations in clefting.

## Introduction

Cleft lip (CL) is one of the most recognizable anomalies and has been the focus of intense clinical and research efforts for many decades. Clefts of the lip can occur with or without clefts of the secondary palate, and collectively represent one of the most common birth defects in humans, occurring with an average global prevalence of 1 in 700 live births (Dixon et al., 2011; Leslie and Marazita, 2013). Yet, pediatricians, surgeons, speech pathologists, nutritionists, geneticists, and developmental biologists often interpret the term “cleft lip” differently. Much of this undocumented confusion resides in the differing level of granularity with which each person considers the disorder. The geneticist might focus on discernible phenotypic differences and heritability, while the surgeon concentrates on appearance and function, and the developmental biologist centers on gene expression and tissue morphogenesis. The use of imprecise terminology in the literature as well as the growing appreciation of the spectrum of phenotypes that are encompassed by this term adds to the confusion. For example, the term “cleft lip” could refer to variable combinations of defects involving the upper lip, the anterior segment of the maxillary alveolar ridge, and/or the portion of the hard palate anterior to the incisive foramen, that is, any phenotype having an embryologic basis as a perturbation of the growth and/or fusion of the medial nasal process with the paired maxillary and lateral nasal processes.

The benefits of describing cleft phenotypes with detail, accuracy, and reproducibility have been well-described (Leslie and Marazita, 2013). Studies using population-level data have detected significant associations between subclasses of cleft types and specific genomic regions. Such subclassification is also likely to be critical for identifying and understanding environmental contributions to clefting and resolving issues related to the optimal surgical approaches for CL repair. For example, children with lip or labioalveolar (upper lip and alveolus) clefts typically undergo initial surgical reconstruction in infancy with a varying number of revision surgeries. Despite the fact that surgical repair is the standard of care for individuals with overt clefting, there is still insufficient data to identify optimal approaches to improve long-term outcomes for specific subphenotypes (Semb et al., 2005a,b; Shaw et al., 2005; Long et al., 2011; Russell et al., 2011). Although the surgical technique and surgeon's level of experience can vary among cleft centers and among experienced surgeons within the same Center, the diversity in clinical outcomes is likely influenced by the lack of understanding of the impact of phenotypic and etiologic variability.

With the need for large clinical, etiologic, and outcome studies, especially those that are multi-center or multi-national in nature, the use of standardized and detailed phenotypic classification is recognized and urged (Allanson et al., 2009a,b; Carey et al., 2012; Cox et al., 2013). In 1953, Pruzansky wrote, “Most classifications that have appeared in the literature are insufficiently descriptive and arbitrarily reflect the clinical interest of a single professional group without providing universal intelligibility” (Pruzansky, 1953). Unfortunately, since then many more cleft classification systems have been described and adopted in different settings, with still none universally accepted (Mooney, 2008). The use of discordant systems in investigations related to clefting, as with any other etiologically complex and variable phenotype, makes it challenging to interpret and integrate study results and perform analyses over pooled data from published studies. In addition, misuse of terminology and the use of imprecise or poorly defined terms further complicate and impede integration efforts, especially for studies reliant on data generated by researchers from different disciplines.

The goal of this article is to help clarify the similarities and differences among the different classification systems to facilitate integration and standardization of CL data. We describe our comprehensive evaluation of more than 20 systems (or modifications thereof) for their ability to classify CL. We assess each system for the level of detail it provides and its ability to distinguish different clinical presentations and build on prior reviews by providing figures and tables that show how the different systems relate to each other. Such explicit comparisons should allow clinicians and researchers to more easily relate patient data classified according to different systems, and in the longer term, pave the way for creation of a computable framework (an ontology) for relating the different classifications in an information retrieval system.

We deliberately avoid recommending use of a given system or creating an all-inclusive classification system. While such a universal system would have its advantages, history tells us that individuals will continue to use the system that best suits their operational needs based on practical limitations (e.g., access to sonography equipment or personnel to identify or define non-overt phenotypes), time constraints, or the fact that a simpler system meets the immediate needs of the research question being pursued. Thus, our goal in this study is to explicitly represent how each system captures the full phenotypic variability encompassed by the diagnosis of CL on the basis of morphological, developmental, and pathogenic properties as well as the etiological attributes responsible for the heterogeneity of CL phenotypes. At the minimum, this detailed comparison should enable researchers to better appreciate the limitations and challenges associated with using disparate classifications systems, and in the longer term, the resulting ontology should be of great utility for inter-center studies and population-level genetic investigations.

## Methods

### Definition and purpose

For the purposes of this article, the term “cleft lip” includes clefts of the upper lip that involve the upper lip independent of whether the maxillary alveolus is also affected. This definition is based primarily on an anatomical perspective, which serves as the foundation for ontological representation of structures and pathologies, such as that promoted by the National Institute of Dental and Craniofacial Research's FaceBase program to support the craniofacial research community (Hochheiser et al., 2011; Brinkley et al., 2013a,b). Thus, the variable phenotypes encompassed by the term “cleft lip” for this article include overt clefts of the upper lip (both complete and incomplete, with or without Simonart's or Simon's bands) (Mulliken and Schmidt, 2013), and defects in the orbicularis oris muscle. While clefts of the upper lip and the maxillary alveolus may share a common developmental origin, we have limited our assessment in this article to the upper lip. As proof of concept, we sought to determine whether the morphological and/or developmental classification by the different systems can provide sufficient information to ascertain whether common attributes in each system are able to be correlated and the disparities between them reconciled.

### Identification of CL classification systems

We focused our investigation on classification systems that included the broad definition of “cleft lip,” that is, those involving structures anterior to the incisive foramen only. To identify the different CL classification systems, we started with the systems utilized in our centers and others familiar to us based on our knowledge and expertise in the field, and then supplemented this by performing a PubMed search to identify additional systems using combinations of the following terms: orofacial cleft, CL, unilateral, bilateral, classification, description, systems, and phenotype. We reviewed both current and historical peer-reviewed literature and texts that provided details regarding classification of CL. In addition, bibliographies within these sources were used to identify further articles for review. We assessed all identified systems for how they defined distinct subgroups of labiopalatal clefts and the specific terminology employed for and within each classification group. The similarities and differences between these systems were then summarized and visually represented where possible. We appreciate that some systems have not been included in this review. This decision was made based on the similarity of some systems to others already assessed in this study.

### Preparation and classification of representative CL images

Images of unrepaired unilateral and bilateral clefts of the upper lip were selected from our craniofacial clinic photographic repository, which includes images acquired using a standardized photographic protocol. Photos were cropped to focus on the nose and lip, and used to create the representative illustrations included in the figures and tables. A subset of these illustrations was then classified using several of the described CL classification systems to enable direct comparison between systems and their relationships to be identified. Three of the authors (Kathie H. Wang, Carrie L. Heike, Timothy C. Cox) coded the images using representative classification systems, and these were then reviewed by two plastic surgeons (Raymond W. Tse, Craig B. Birgfeld).

## Results

Over 20 classification systems were included in our review. As outlined below, most systems allowed for classification of CL independent of clefting of the maxillary alveolus and secondary palate, while other systems classified CL based on patterns of both pre- and post-incisive foramen clefts.

### Classification systems based on patterns of labiopalatal clefting

Systems designed to capture patterns of cleft phenotypes based on morphology, embryogenesis, and pathogenesis typically include distinct configurations of, and relationships among, clefts of the upper lip, alveolus, and the hard and soft palate, as shown in Table 1. For example, the Davies–Ritchie (Davis and Ritchie, 1922) and Iowa (Hanson and Murray, 1990) systems allow for classification of either unilateral or bilateral CL in distinct groups (e.g., “Group 1” in both systems), while those of Veau, Pruzansky, Ross, and Johnston, and ICPR (Veau, 1931; Pruzansky, 1953; Ross and Johnston, 1972; Millard, 1976) incorporate CL into categories that include clefts of the alveolus and the secondary palate (hard, and/or soft palate) (see Table 1). None of these systems were designed to provide detail specifically about CL characteristics. For example, most do not distinguish between incomplete and complete CL, while those that do so differ in their reliance on either the “unaffected” or “more severely affected” side when selecting the appropriate classification. These systems therefore do not distinguish between variable patterns with incomplete clefts of the lip, or asymmetric bilateral clefts of the lip.

**Table 1:**
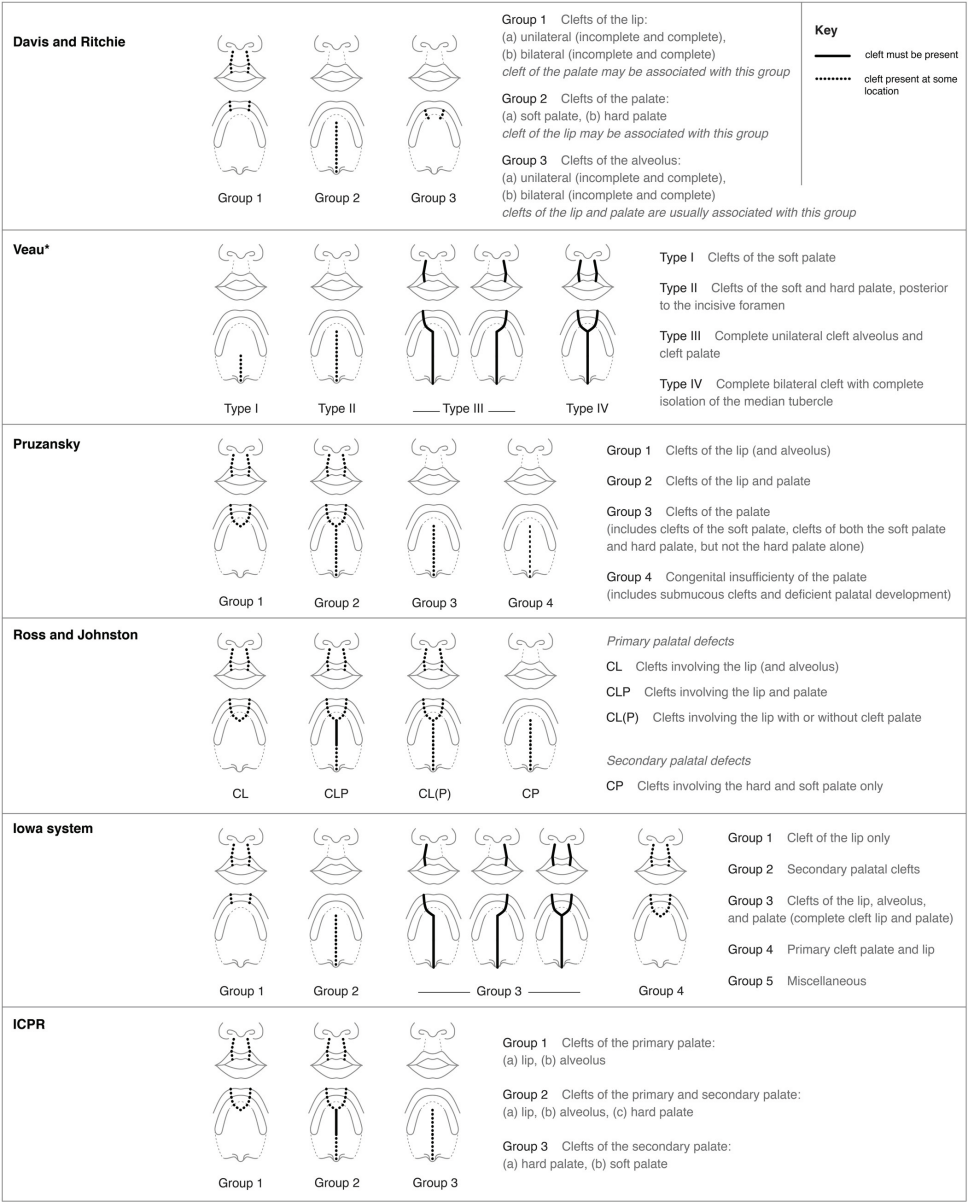
**Comparisons among classification systems based on patterns of labiopalatal clefting**.

^*^Interpretation of this system was based on secondary descriptions (Mooney, 2008).

### Classification systems providing distinction between and among unilateral and bilateral clefts

We identified several systems that allowed for more detailed descriptions of clefts of the upper lip, independent from the accompanying phenotypes of the maxillary alveolus, hard and soft palate. Most systems include specific codes to represent unique cleft types but with different levels of granularity. Table 2 compares the classification properties used by different systems, noting that some systems were omitted on the basis of their complexity and hence difficulty to display in a comparable format. The London Dysmorphology Database (LDD), also referred to as the Winter-Baraitser Dysmorphology Database (www.lmdatabases.com/about_lmd.html), and International Classification of Disease (ICD)-10 (http://www.who.int/classifications/icd/icdonlineversions/en/) systems classify clefting based only on a general anatomical location, hence a generic classification for “cleft lip.” The RPL system (Schwartz et al., 1993) and Kernahan's Striped “Y” coding scheme (Kernahan, 1971) both provide the same general category while specifying the laterality attributes (left/right clefts). The earlier ICD version, ICD-9 (http://www.cdc.gov/nchs/icd/icd9cm_addenda_guidelines.htm), Elsahy (Elsahy, 1973), Santiago (Santiago, 1969), Spina (Spina, 1973), LAHSHAL (Kriens, 1989), and LAHSN (Koch et al., 1995) classification systems, some of which are derivatives of the aforementioned systems, describe the extent of clefting as degrees of completeness and therefore classify clefts as either incomplete/partial/subtotal clefts of the upper lip, or complete or total clefts of the upper lip as illustrated in Table 2A. The LAHSN system further elaborates on the severity of all single or combined malformations based on the extent of the defect in transverse, vertical, and sagittal directions (Koch et al., 1995). Onizuka's system, despite incorporating subclassifications of the lip into fourths, does not utilize these subclassifications in the overall cleft categorization (Onizuka et al., 1991). As such, we included the system in Table 2A. Except for ICD-9 and Onizuka's system, these systems declare right or left occurrence. The LAPAL system (Liu et al., 2007), Natsume's system (Natsume et al., 1984), Koul's Expression system (Koul, 2007), the modified Kernahan's Striped “Y” systems (Friedman et al., 1991; Smith et al., 1998), and Harkins' system (Harkins et al., 1962) divide the lip into halves or thirds, each with different code sets, as illustrated in Table 2B. However, most of these systems do not provide specific details on how the halves or thirds should be determined, for example by anatomical landmarks or quantitative measurements. Only Onizuka's system specifies that incomplete clefts are measured based on the whole lip, although the exact location of measurement of the whole lip is not explained (Onizuka et al., 1991). On the other hand, the Clock diagram subclassifies CL severity based on actual measurements of the width of the clefts and degrees of rotation of Cupid's bow rather than the vertical severity of clefts (Rossell-Perry, 2009). The Ortiz-Posadas system also incorporates measurements of cleft size as well as a variety of other subjective measures (Ortiz-Posadas et al., 2001). Because of the complexity in subclassifying individual cleft phenotypes with these two systems, they were not included in Table 2.

**Table 2:**
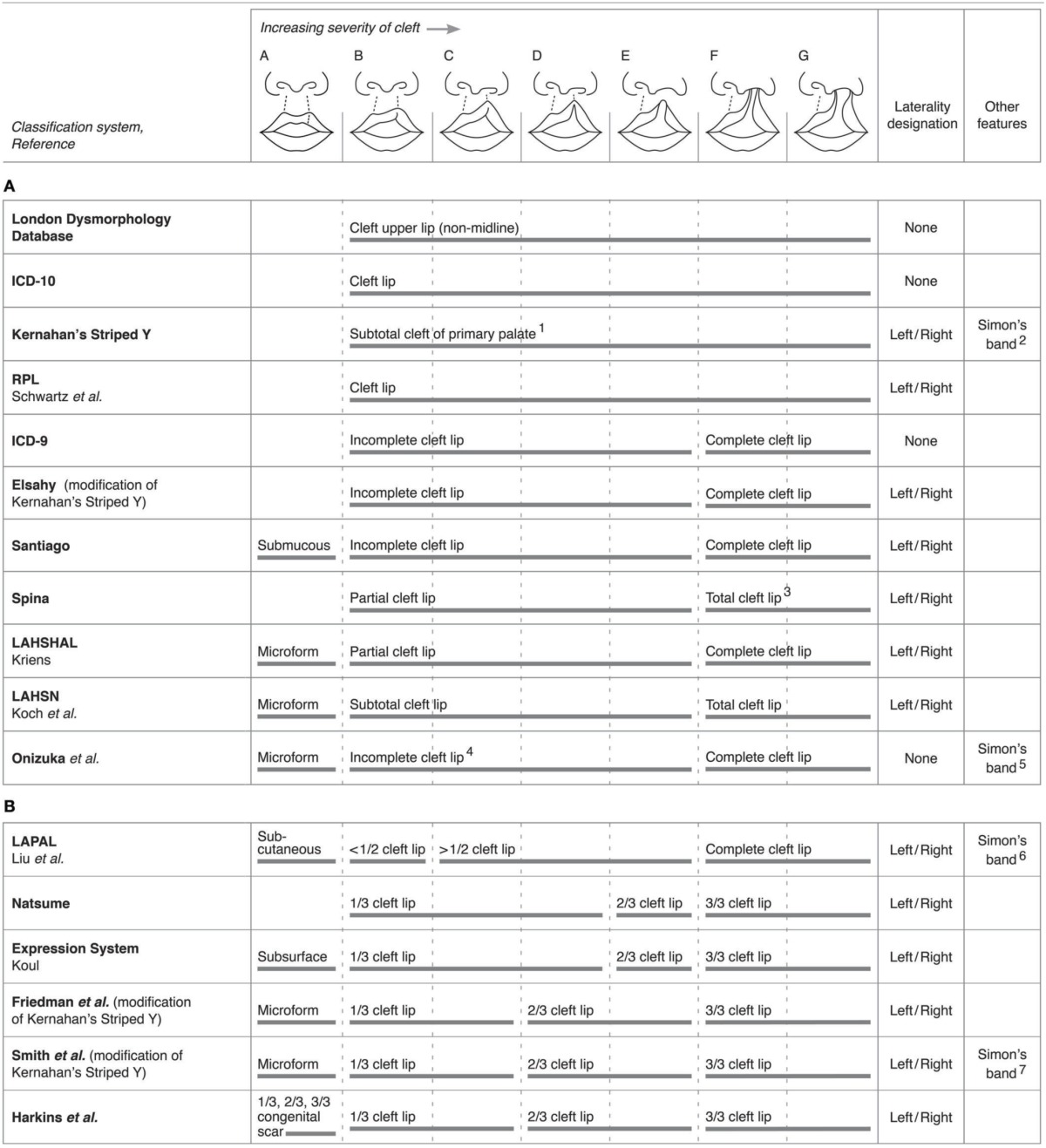
**Comparisons among systems for their ability to classify varying degrees of severity of unilateral clefts of the lip**.

^*1*^Does not distinguish between complete and incomplete clefts unless considering the rest of the primary palate.

^*2*^Simon's band to be indicated with cross-hatching on the “Y” diagram.

^*3*^Total cleft when the cleft reaches the alveolus.

^*4*^Clefts up to 1/4,1/2, and 3/4 of the lip are grouped as incomplete clefts.

^*5*^A complete cleft with a Simon's band is included in the same category as a complete cleft without a Simon's band.

^*6*^Simon's band included in the same category as a > 1/2 cleft lip.

^*7*^Simon's band categorized separately from incomplete and complete cleft lips.

Surprisingly, some classification systems do not accommodate comprehensive characterization of bilateral CL, but many of those that do are illustrated in Table 3. The ICD-10 system, which contains separate codes for unilateral and bilateral clefts of the upper lip, and the RPL system (Schwartz et al., 1993) do not allow for more detailed characterization of the degree of bilateral clefting nor presence of asymmetry. Most systems, however, accommodate the characterization of bilateral clefts with independent classification of the left and right clefts, describing degrees of completeness in halves (< 1/2, > 1/2) or thirds (1/3, 2/3, 3/3) on both sides of the lips, except for the LAHSN system. The Clock diagram classifies bilateral clefts in a manner similar to how it classifies unilateral clefts, i.e., with measurements of cleft width, but also includes divisions of the columella as well as prolabium length for bilateral rankings, and a variable to capture asymmetry between the right and left sides (Rossell-Perry, 2009). Again, it was not included in the table due to its complexity in ranking.

**Table 3:**
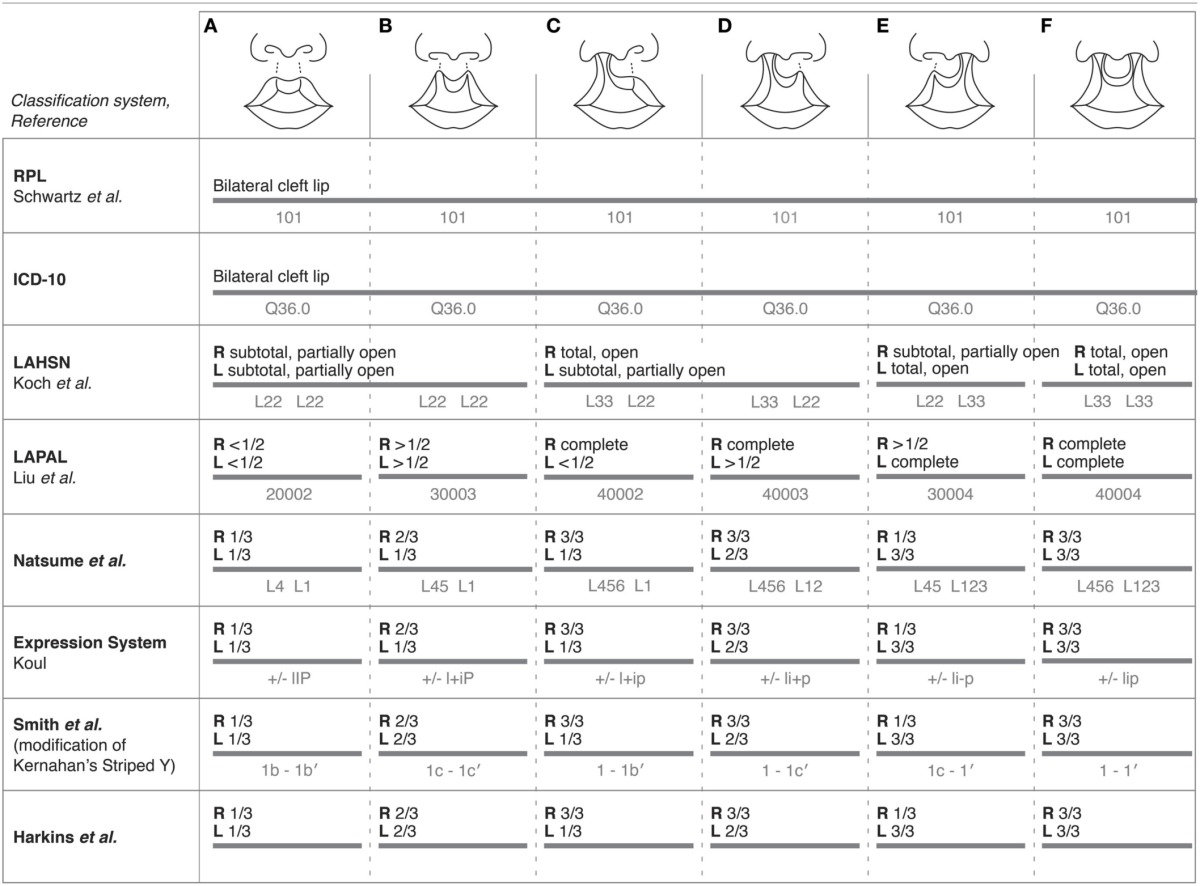
**Comparisons among select systems for their ability to classify and code for characteristics of bilateral clefts of the lip with varying degrees of severity**.

As illustrated in Table 3, a variety of systems utilize unique coding schemes with numerical and/or alphabetical combinations to subclassify clefts. The Expression system classifies unilateral and bilateral clefts using a combination of symbols and capitalization of “lip”; for example, a bilateral cleft not crossing the vermilion border would be written as ± lIP, whereas a right unilateral incomplete cleft crossing the vermilion border would be written as + liP (Koul, 2007). Regardless of whether or not systems subclassify clefts the same way, i.e., by splitting the lip into thirds, each system still uses a distinct coding system to document a cleft.

### Characterization of complete and incomplete CL

Systems also differ in their ability to distinguish between the general categories of “complete” and “incomplete.” In some cases this is related to the level of granularity provided by the system, while more typically it is related to either ambiguity in definition of cleft characteristics or a reliance on subjective determination of the extent of clefting and not on actual anatomical measurements or landmarks. The ICD-9, Friedman's and Smith's modifications of Kernahan's Striped “Y,” LAPAL, LAHSHAL, Santiago, and Ortiz-Posadas do not provide definitions for a complete or total cleft [http://www.cdc.gov/nchs/icd/icd9cm_addenda_guidelines.htm; (Santiago, 1969; Kriens, 1989; Friedman et al., 1991; Ortiz-Posadas et al., 2001; Liu et al., 2007)]. The Elsahy system provides the simplest and clearest definition for a complete cleft—that is, those that include the nostril floor (Elsahy, 1973). Harkins', Natsume's, and the Expression systems define a complete cleft in a similar manner, where the cleft is described to extend *into* the nostril, nasal cavity or proboscis, respectively (Harkins et al., 1962; Natsume et al., 1984; Koul, 2007). Spina characterizes a total cleft as one that reaches the alveolar arcade, but no specific definition of the alveolar arcade is given (Spina, 1973). By contrast, the LAHSN system defines a complete cleft as one where there is “complete interruption of functional tissue (bone, muscle, or cartilage) with no physiologic function” (Koch et al., 1995). We summarize these definitions in Table 4. Additionally, it is unclear how some of these systems would classify a patient with a Simon's band (previously and widely referred to as a Simonart's band) (Mulliken and Schmidt, 2013), which is a residual bridge of epithelial tissue superficially connecting the lip or alveolus. Kernahan's Striped “Y” documents a cleft with a Simon's band through cross-hatching on the anterior limb of the “Y” (Kernahan, 1971). Onizuka's system does acknowledge complete clefts with and without a Simon's band, but groups them both into a fourth degree complete cleft (Onizuka et al., 1991). Smith's modification of Kernahan's Striped “Y” classifies a lip with a Simon's band as an incomplete cleft (Smith et al., 1998). The LAPAL system groups clefts with a Simon's band into the same category as clefts > 1/2 of the lip (Liu et al., 2007).

**Table 4:** Examples of variation in definitions for common terms used to describe cleft lip^*^.

**System references**	**Complete cleft lip**	**Incomplete cleft lip**	**1/3 incomplete cleft lip**	**Most minor form of cleft lip**
Modified striped “Y” Elsahy, 1973	Includes nostril floor			
Expression Koul, 2007	Extends into proboscis		Does not cross vermillion border (lip indentation)	Subsurface cleft
Modified striped “Y” Friedman et al., 1991			Overt cleft of 1/3 of the vertical dimension of the lip	Microform cleft: Congenital scar (subcutaneous cleft), or notch in vermillion border
Harkins et al., 1962	Extends into nostril			Congenital scar
LAHSHAL Kriens, 1989				Microform cleft
LAHSN Koch et al., 1995	Complete interruption of functional tissue	Insufficient function of affected layers		Microform cleft: Functional muscle for lip unaffected
Onizuka et al., 1991	With or without Simon's band	Cleft up to 1/4, 1/2, or 3/4 of whole lip		Microform cleft: cleft lip nose without lip deformities, notch of vermilion free margin, notch or vermilion border, or striae of lip
LAPAL Liu et al., 2007				Subcutaneous cleft: Usually includes minor cleft in vermillion
Natsume et al., 1984	Extends into nasal sill based on diagram		Does not cross vermillion border (based on diagram)	
Santiago, 1969				Submucous cleft
Modified striped “Y” Smith et al., 1998				Microform cleft
Spina, 1973	Reaches alveolar arcade			
Yuzuriha and Mulliken, 2008				Mini-Microform cleft: Discontinuous vermillion-cutaneous junction, level Cupid's bow peaks, notched free mucosal margin, variable muscular depression

* Definitions identified from text and/or interpretation of figures included in the respective reference.

Three systems offered quantitative measurements in the assessment of complete cleft phenotypes (Ortiz-Posadas et al., 2001; Yuzuriha and Mulliken, 2008; Rossell-Perry, 2009). Along with visual guides, the Clock diagram measures the angle of Cupid's bow and the width of the cleft(s) to rate the degree of completeness and severity (Rossell-Perry, 2009). However, the Clock diagram does not strictly subclassify complete clefts, but simply regards a severe cleft as one that has no nasal columella, a prolabium less than or equal to 1/3 of the lateral segment length, and a cleft of the primary palate wider than 15 mm (Rossell-Perry, 2009). Yuzuriha and Mulliken, in contrast, include measurements of the distance between the vermilion-cutaneous point and the normal Cupid's bow peak together with visual comparisons of anatomical landmarks on the lip and nose to define degrees of completeness (Yuzuriha and Mulliken, 2008). Because the specific features qualifying for each category of the Yuzuriha and Mulliken system were outside the spectrum of our visual representations, we also did not include this classification system in Table 2. The system described by Ortiz-Posadas and colleagues assigns graded scores to each feature, based on cleft width measurements of each side independently, to produce an overall composite score of the cleft complexity (Ortiz-Posadas et al., 2001). This is complemented by a subjective descriptive assessment of the aesthetics of the lip and nose using the categories “yes,” “almost,” “barely,” or “no”; the length of the columella using “normal,” “almost,” “barely,” or “absent”; and the width of the nasal base as “greater,” “normal,” and “smaller” (Ortiz-Posadas et al., 2001).

In addition to variation in definitions of “complete,” definitions of incomplete cleft classifications also vary in specificity. The LAHSN system vaguely defines an incomplete or “subtotal” cleft as one where the “functional tissue” is partially affected and hence, there is “insufficient function of affected layers” (Koch et al., 1995). The LAPAL system subdivides incomplete clefts into those that are less than or greater than 1/2 of the lip, although definitions of the points defining the full length and thus in turn the midpoint are not provided (Liu et al., 2007). The systems that divide the lip into three regions appear to differ in the way in which these divisions are defined. For instance, the Natsume (Natsume et al., 1984) and Expression (Koul, 2007) systems define clefts that do not cross the vermillion border as “1/3,” whereas the Harkins, Smith's modification of Kernahan's Striped “Y,” and the Ortiz-Posadas systems do not define “1/3” (Harkins et al., 1962; Smith et al., 1998; Ortiz-Posadas et al., 2001). Finally, the Onizuka system divides incomplete clefts into fourths with respect to the whole lip (Onizuka et al., 1991). We provide these anatomic definitions in Table 4.

**Figure 1:**
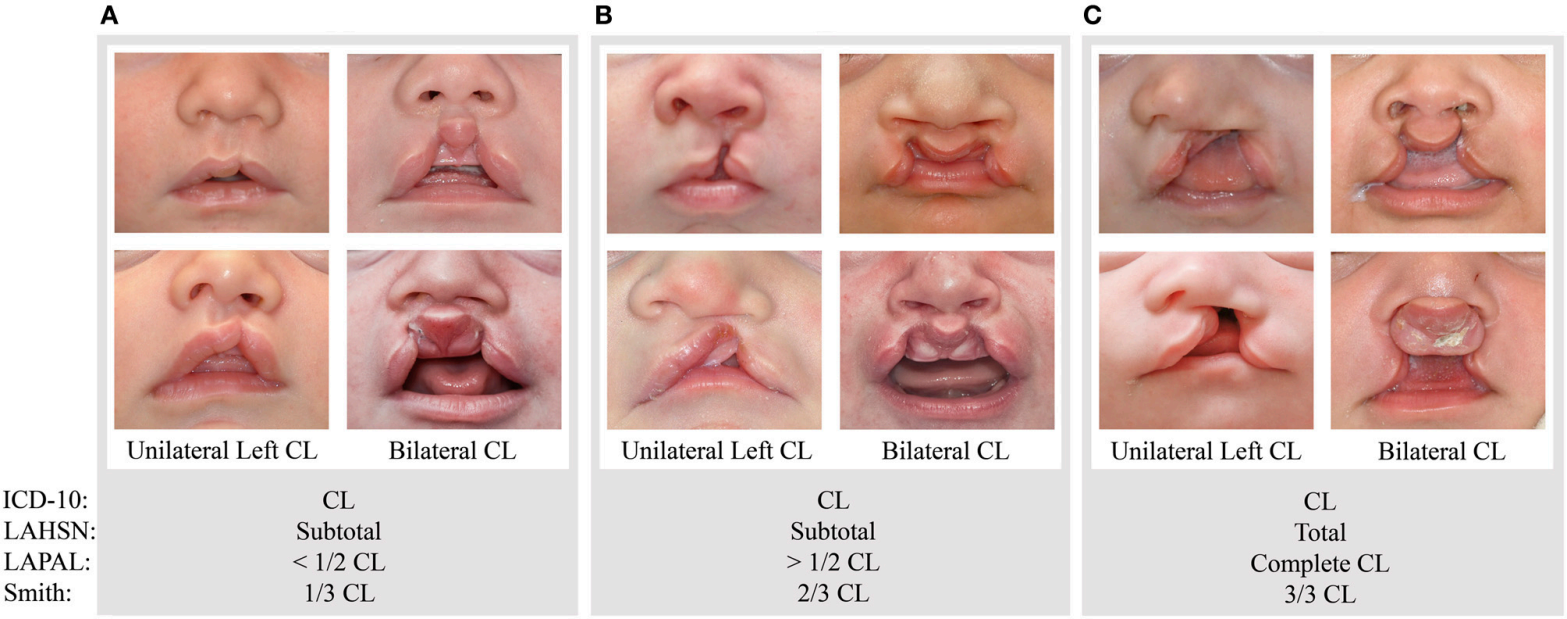
**Example rankings of varying unilateral left and bilateral phenotypic presentations based on subjective interpretations of the ICD-10, LAHSN (Koch et al., 1995), LAPAL (Liu et al., 2007), and Smith's modification of Kernahan's Striped “Y” (Smith et al., 1998)**. A cleft from Group **(A)** would be classified as a unilateral or bilateral CL, subtotal cleft, < 1/2 CL, or 1/3 CL, respectively. A cleft from Group **(B)** would be classified as a unilateral or bilateral CL, subtotal cleft, > 1/2 CL, or 2/3 CL, respectively. A cleft from Group **(C)** would be classified as a unilateral or bilateral CL, total cleft, complete CL, or 3/3 CL, respectively.

To investigate how these different systems of division impacted classification, we used each system to categorize the degrees of incompleteness on a set of images of various types of CL. For simplicity, we classified them according to the criteria used in four systems: the ICD-10, LAHSN, LAPAL, and Smith systems (http://www.who.int/classifications/icd/icdonlineversions/en/; Koch et al., 1995; Smith et al., 1998; Liu et al., 2007). For example, the most severe set of bilateral clefts differ in the size of the prolabium, but they receive the same rankings under all four systems (Figure 1 and Table 3). Likewise, the two most severe unilateral CLs both receive the same rankings as well (Figure 1), although they differ in the width of the clefts—which would be captured by the Clock diagram.

### Classification of mild or subclinical CL

Less than half of the CL classification systems address more subtle cleft-related presentations. Some systems refer to this milder form of clefting as a “congenital scar” or “subcutaneous cleft,” while others use the term “microform” to represent the mild or subclinical manifestation of a pathology (Table 4). Hereafter, we will refer to this phenotype as a forme fruste, which incorporates these terms in the same entity. The ICD-9, ICD-10, LDD, Kernahan's striped “Y” (Kernahan, 1971), the RPL system (Schwartz et al., 1993), Elsahy's modification of Kernahan's Striped “Y” (Elsahy, 1973), Natsume's system (Natsume et al., 1984), and Spina's system (Spina, 1973) do not capture forme fruste in a classification, nor do they include subclinical presentations such as defects of the orbicularis oris muscle (OO defect). However, it is now appreciated that such defects, which may be the most minor form of clefting, are more prevalent in so-called “unaffected” family members of an individual with an overt orofacial cleft (Dixon et al., 2011). Identifying and recording such subtle defects is not only important for research purposes but also is a critical piece of information for the clinical geneticist when considering risk for subsequent pregnancies (Leslie and Marazita, 2013). Such variants are labeled in very different manners among systems. Harkins refers to a forme fruste as a congenital scar, and is the only system to divide it into thirds (Harkins et al., 1962). The LAPAL and Expression systems classify minor clefts as “subcutaneous” and “subsurface” clefts, respectively, but they offer no definitions for these terms and it is not clear whether they refer only to OO defects without observable external manifestations on the skin or mucosa (Koul, 2007; Liu et al., 2007). The Santiago system defines this defect as a submucous cleft (Santiago, 1969). The LAHSN system defines a microform cleft as a cleft where the “functional” tissues of the lip are unaffected upon examination in the transverse, vertical, and sagittal directions (Koch et al., 1995). Friedman's modification of Kernahan's Striped “Y” defines a microform cleft as a congenital scar or subcutaneous cleft, or a notch in the vermilion border (Friedman et al., 1991). Smith's modification of Kernahan's Striped “Y” (Smith et al., 1998) and the system of Ortiz-Posadas (Ortiz-Posadas et al., 2001) do not define their category of “microform,” while Yuzuriha and Mulliken (2008) define a microform cleft as a notch less than 3 mm above Cupid's bow peak. Any disruption of the vermilion-cutaneous junction without elevation of the bow peak is considered a mini-microform cleft (Yuzuriha and Mulliken, 2008). The Onizuka system classifies microform clefts as first degree clefts (CL nose without lip deformities), or second degree clefts (traced CLs), which include notches of the vermilion free margin, notch of the vermilion border, or striae of the lip (Onizuka et al., 1991). Because of a lack of definitive information on the description of forme fruste or microform cleft in some of the systems and the disparate definitions between those that do describe this type of cleft, no consensus has yet been achieved on its precise classification.

### Variation in cleft images

Several systems include visual illustrations of clefts to demonstrate examples of their classification methods. For example, Harkins includes schematics of various unilateral and bilateral clefts, but the illustrated clefts do not cross the vermillion border until they fully extend into the nasal sill (Harkins et al., 1962). This contradicts the definitions from the Natsume and Expression systems, where a cleft greater than 1/3 of the lip must cross the vermillion border (Natsume et al., 1984; Koul, 2007). Yuzuriha and Mulliken include detailed illustrations of minor-form, microform, and mini-microform clefts (Yuzuriha and Mulliken, 2008), but all three include interruptions of the vermillion border, which classify them all as 2/3 clefts under the Natsume and Expression systems (Natsume et al., 1984; Koul, 2007). Several other systems also include illustrations, all in varying formats and amount of detail. The RPL, LAPAL, ICD-9, ICD-10, and LDD systems do not include images or illustrations to further expand upon their classification systems (Schwartz et al., 1993; Liu et al., 2007; http://www.cdc.gov/nchs/icd/icd9cm_addenda_guidelines.htm; http://www.who.int/classifications/icd/icdonlineversions/en/; www.lmdatabases.com/about_lmd.html).

## Discussion

Consistent and standardized terminology for the detailed symbolic representation of phenotypes is a necessity for effective large clinical, etiologic, and outcome studies (Allanson et al., 2009a; Carey et al., 2012; Cox et al., 2013). However, many systems have been created to classify cleft phenotypes using different formats and terms. These systems have been developed to suit operational needs, such as clinical billing, surgical planning, outcome assessment, and basic etiologic research (Mooney, 2008). Each has its own merits, but none sufficiently encompass all the subphenotypes and no system has been universally adopted. Instead, most clinical and research teams use different systems likely because their specific goals are unique and the system(s) chosen sufficiently meet those needs. However, in many cases, these systems do not scale up to accommodate new advances or support new discoveries that can be gained through interaction and interoperability with other efforts.

The purpose of this review was not to create a new clinical classification system, but rather to examine and compare existing cleft classification systems, assess their utility and determine if and how these systems can be related to each other to maximize integration and interpretation of different studies. We have identified the similarities and discrepancies among classification systems for subcategorizing unilateral and bilateral clefts of the upper lip. The broad categories used by systems such as ICD-10 and LDD are intended for general coding of diseases only. In contrast, other systems represent clefts at varying levels of granularity in attempts to categorize affected individuals in terms of anatomical manifestation, but often without any reference to etiology.

Our analysis of cleft classification systems is of particular relevance to research focused on identification of the etiological basis of the disorder and also on improving clinical outcomes. In the clinic, the use of, or lack of familiarity with, disparate classification systems may cause miscommunication when studying and treating specific types of clefts. Similarly, in research, the use of disparate classification systems by different Centers participating in a national or international-scale study may significantly confound interpretations of data and unduly skew the significance of any findings. Furthermore, the inability to precisely define and subclassify phenotypes is widely believed to limit the power of population-based genetic and epidemiological studies. This has been amply highlighted by recent genome-wide association studies (Dixon et al., 2011; Leslie and Marazita, 2013). That said, many epidemiologic investigations still typically classify individuals with clefts of the lip and palate into a single case group, or divide subgroups into categories of CL with or without cleft palate (CL± P), and/or syndromic or non-syndromic clefting. Greater specificity in subclassification of phenotypes in clefting is likely to enhance the power for detecting genotype-phenotype and gene-environment correlations underlying the susceptibility to, and defining the severity of, orofacial clefts. Refined classification systems would also likely enhance treatment outcome studies and allow for interpretation of treatment outcomes across studies.

In evaluating the many existing systems, we have found that most do not provide sufficiently detailed definitions as to unambiguously assign some cleft presentations to a specific subclass. Some systems exclude non-overt phenotypes or fail to distinguish those clefts involving just the lip vs. those with alveolar involvement. However, regardless of the classification system selected, the figures and tables in this article should, at minimum, allow investigators to appreciate how to better integrate phenotypic data reported by different groups regardless of the classification system employed.

We acknowledge that there are some limitations in our approach used to compare systems. We elected to use ratings on standardized photographic image data of the lip and nose and representative illustrations rather than in-person assessments performed during a clinical visit. Although photographs may not be sufficient for comprehensive classification using the Clock diagram and others requiring measurements, these images serve as an inexpensive high-resolution record of the raw data and are routinely available to most clinical and research teams. We also used select images from our own clinical repository to represent the more common forms of clefting and the variation in classification among systems, although we did not include all known variations of unilateral and bilateral clefts of the lip. We also focused our efforts on clefting of the lip only and did not consider combinations of clefts of the lip, alveolus, hard and soft palate in our comparison of the different systems, even though some of the selected systems do provide subclassification based on presentations involving these orofacial components. This decision was made in an effort to reduce the complexity and variability of cleft presentations and simplify interpretations of the different systems in a practical setting. We did not distinguish between phenotypic variation in syndromic and non-syndromic clefting. Furthermore, characteristics of the nose were not included in our categorization because many of the systems we examined also did not rely on such characteristics. We also note that reliability estimates have not been published for most of the systems we included in our comparisons; however, high inter and intra-rater reliability is an essential feature of any classification system that will be used in clinical practice and research and a critical factor to consider as we move toward development of consensus for data standards regarding CL. Furthermore, ongoing clinical training in the documentation of CL (and indeed cleft palate) classifications should also be considered. The Crux Cleft Palate Database (DF) is an example of a computerized system that includes a “training” module with 54 different descriptions of orofacial clefting, involving various combinations of the segments of the lip, alveolus and hard and soft palates. New users of the Crux Database are required to be able to reproduce all given cleft descriptions, e.g., “Bilateral (complete right, incomplete left) CL with complete cleft of the alveolus with Simon's band on the right” above an accuracy level of 98% (Fitzsimons, 2006). Indeed, the need to be able to reliably and accurately reproduce the documentation of a given cleft condition from an agreed description may be a useful intermediate step before training cleft team members to document and classify patient images/photographs. Nevertheless, by highlighting the limitations and challenges associated with using disparate classifications systems, it is hoped that this assessment and comparison of each system will promote more discussion and cooperation in standardizing the classification of orofacial clefts.

To further facilitate this and to integrate such systems into a broader framework of craniofacial data, a specific ontology—the Ontology of Craniofacial Development and Malformation (OCDM)—is being developed as part of the NIDCR-funded research network, FaceBase (https://www.facebase.org). The purpose of FaceBase is to provide diverse but standardized data to the craniofacial community and to facilitate collaboration among investigators to advance craniofacial research. The goal of the OCDM is to provide a unifying framework to represent and standardize the set of terms and relationships used to capture different forms of craniofacial data, including clinical data, within FaceBase and integrate data types to maximize their utility and accessibility (Hochheiser et al., 2011; Brinkley et al., 2013a,b). The findings from this study are one of the first steps to integrate clinical data into this network. It is anticipated that these and other concerted efforts by the craniofacial community will underpin more efficient and effective data collection to advance research into and treatment of patients with craniofacial conditions such as orofacial clefting.

### Conflict of interest statement

The authors declare that the research was conducted in the absence of any commercial or financial relationships that could be construed as a potential conflict of interest.
